# *Aeromonas hydrophila* induction method in adult zebrafish (*Danio rerio*) as animal infection models

**DOI:** 10.14202/vetworld.2023.250-257

**Published:** 2023-02-09

**Authors:** Dahliatul Qosimah, Sanarto Santoso, Maftuch Maftuch, Husnul Khotimah, Loeki Enggar Fitri, Aulanni’am Aulanni’am, Lucia Tri Suwanti

**Affiliations:** 1 Doctoral Study Program in Medical Science, Faculty of Medicine, Universitas Brawijaya, Malang, Indonesia; 2 Laboratory of Microbiology and Immunology, Faculty of Veterinary Medicine, Universitas Brawijaya, Malang, Indonesia; 3 Laboratory of Microbiology, Faculty of Medicine, Universitas Brawijaya, Malang, Indonesia; 4 Laboratory of Fish Diseases, Faculty of Fisheries and Marine Sciences, Universitas Brawijaya, Malang, Indonesia; 5 Laboratory of Pharmacology, Faculty of Medicine, Universitas Brawijaya, Malang, Indonesia; 6 Laboratory of Parasitology, Faculty of Medicine, Universitas Brawijaya, Malang, Indonesia; 7 Department of Chemistry, Faculty of Sciences, Universitas Brawijaya, Malang, Indonesia; 8 Department of Veterinary Parasitology, Faculty of Veterinary Medicine, Airlangga University, Surabaya, Indonesia

**Keywords:** *Aeromonas hydrophila*, animal model, hematological profile, survival rate, zebrafish

## Abstract

**Background and Aim:**

Zebrafish are frequently used as model organisms in scientific research as their genes mirror those of humans. *Aeromonas hydrophila* bacteria can infect humans and animals, mainly fish. This study aimed to identify the concentration and route of *A. hydrophila* infection in adult zebrafish. Zebrafish had been used as a challenge test by analyzing their hematological profiles, blood glucose levels, and survival rates.

**Materials and Methods:**

Induction of cell supernatant free (CSF) from *A. hydrophila* bacteria in adult zebrafish was carried out via bath immersion (BI), intraperitoneal injection (IPI), intramuscular injection (IMI), and healthy zebrafish as a control (C). The bacterial concentrations were 10^7^, 10^9^, and 10^11^ colony-forming units (CFU)/mL. At 24 h post-infection, the outcomes of infection were evaluated based on survival rates, hematological profiles, and blood glucose levels. A one-way analysis of variance with a confidence level of 95% was employed to examine the data.

**Results:**

In the BI, IPI, and IMI treatment groups, the survival rate of the fish reached a peak of 100%, 22%–100%, and 16%–63%, respectively, compared with the injection technique. In the IMI2 group, a 10^9^ CFU/mL bacterial concentration was determined to correspond to the lethal dosage 50. All infection groups had lower erythrocyte and hemoglobin counts but higher leukocyte counts than the control group. The blood sugar levels of the healthy and infected groups were not significantly different.

**Conclusion:**

The route of *A. hydrophil*a infection through Intramuscular injection with a concentration of 10^9^ CFU/mL indicated a high performance compared to other techniques. This method could be developed as a reproducible challenge test.

## Introduction

Among the various pathogens, bacteria exhibit the greatest potential threat to aquaculture life. *Aeromonas hydrophila* bacteria cause fish to develop epizootic and hemorrhagic ulcerative syndrome and induce pathogenicity in fish and humans [1]. In addition, these bacteria can infect freshwater fish worldwide, causing huge losses due to high mortality rates [2], reaching 80%–100% within 1–2 weeks [3], and high cost. Furthermore, commercially available vaccines are ineffective in controlling the spread of these bacteria due to the complexity of the antigen structure [4].

Zebrafish is an essential species in ornamental fish trade and scientific research [5]. They are frequently used as animal models for the prevention and treatment of diseases. They exhibit genome homology of >80% with humans associated with diseases [6]. At present, zebrafish larvae are widely used as animal models to improve understanding of host and pathogen interactions. There has also been extensive use of zebrafish larvae to describe bacterial pathophysiologies, such as infection with *Mycobacterium marinum*, *Staphylococcus aureus* [6], and *A. hydrophila* [7]. Zebrafish larvae exhibit fast growth, transparent bodies, genetic manipulability [6], and quicker rearing than mammalian models. However, they only have innate immunity, and their adaptive immunity only develops 4–6 weeks after fertilization in the juvenile phase [8]. Scientific information regarding the use of animal models of zebrafish larvae infected with *A. hydrophila* by various routes of infection is available [9], but the use of adult zebrafish is lacking. Macrophage cells perform a metabolic shift to deal with infection, resulting in an increase in glycolysis that triggers antimicrobial and cytokine production [10]. *Salmonella* bacterial infection triggers low nutrient uptake, decreasing blood glucose levels in broiler chickens [11]. The previous research demonstrated that it is possible to identify a new phenomenon related to the relationship between *A. hydrophila* bacterial infection and changes in blood glucose levels in the fish’s body.

Hematological tests and routine diagnostic methods can determine physiological disorders, diagnostic tools, and disease prognosis [12]. As a preliminary step in developing treatments for infectious diseases, it is necessary to examine the concentration of contagious or fatal microorganisms in fish. Based on changes in survival rates, blood profiles, and blood sugar levels, adult zebrafish are expected to be used as animal models of *A. hydrophila* infection.

This study aimed to identify the best concentration and route of infection of *A. hydrophila* in adult zebrafish acting as animal models, which can be used as a challenge test by analyzing the hematological profiles, blood glucose levels, and survival rates.

## Materials and Methods

### Ethical approval

Ethical approval for this study was obtained from the ethics committee (certification no. 139-KEP-UB-2022) of Universitas Brawijaya.

### Study period and location

The study was conducted from 11-07-2022 to 25-07-2022 at the Laboratory of Fish Diseases, Faculty of Fisheries and Marine Sciences, Universitas Brawijaya, Malang, Indonesia.

### Zebrafish preparation

Five-month-old healthy wild-type zebrafish strains were obtained from the Faculty of Fisheries and Marine Affairs, Universitas Brawijaya, Indonesia. The fish spent 3 days adjusting to the environmental conditions and were fed twice a day [13].

Tanks with a volume of 20 L were cleaned and then poured with calcium hypochlorite (Tradeasia, Indonesia) as much as 480 mg/16 L of water [14] as a disinfectant to kill microbes in the water and 240 mg sodium sulfate/16 L of water to neutralize the pH level of the water The containers were rinsed with tap water and sucked out. The fish were maintained at a temperature of 26°C–28.5°C, a pH level of 6.8–7.5, and dissolved oxygen of >6.0 mg/L. This study used 32 fish in a 16 L tank of water for every group [15].

### Preparation of *A. hydrophila* suspension

Gram staining, biochemical properties, and macroscopic examination of colonies were employed to confirm the presence of *A. hydrophila* bacteria. The bacterial suspension was prepared by planting *A. hydrophila* colonies on tryptic soy agar growth media (Merck, Darmstadt, Germany) and incubating them for 24 h at 27°C–28°C. In addition, the bacterial colonies were cultivated in brain heart infusion broth (BHIB; Merck) media for 24 h at an incubation temperature of 27°C–28°C. The bacterial culture was centrifuged at 3075× g for 15 min to obtain pellets/cell-free supernatant (CFS) [16]. By adding 5 mL of physiological saline, the CFS bacteria were resuspended, and optical density was measured using a spectrophotometer (Thermo Fisher Scientific, USA) with a wavelength of 625 nm. A series of dilutions were performed so that the estimated quantity of bacteria was 10^11^ colony-forming units (CFU)/mL.

### Infection treatment of *A. hydrophila*

Infection treatment was through immersion and injection induced *A. hydrophila* bacteria in adult zebrafish. A total of 320 fish were divided into three treatment groups (each group contained 32 fish with three replications) and one control group (n = 32). The treatment consisted of a control group (C); bath-immersed group (BI1, BI2, and BI3) by mixing 160 μL of bacterial suspension at a concentration of 10^11^ CFU/mL in 16 L of water (at final concentrations of 10^5^, 10^7^, and 10^9^ CFU/mL); injection treatment by inserting a 10 μL bacterial suspension (at concentrations of 10^7^, 10^9^, and 10^11^ CFU/mL) intraperitoneal injection (IPI) (IPI1, IPI2, and IPI3) in the abdominal cavity, behind the pelvis, and intramuscular injection (IMI) (IMI1, IMI2, and IMI3) in the muscle in the area near the dorsal fin using a sterile syringe with a volume of 1 mL and a needle size of 34 G (Terumo) [17, 18]. In addition, the fish were observed for clinical signs, and the survival rate and lethal dosage 50 (LD_50_) were determined 24 h after infection during the acute toxicity test.

### Hematological parameter

The fish blood was collected from the caudal artery (*Linea lateralis*) 24 h after the infection was introduced [19] and mixed with 3% ethylenediaminetetraacetic acid anticoagulant [4]. The plasma was then examined, and the blood profile (hemoglobin, erythrocytes, leukocytes, and leukocyte differential) and blood sugar levels were determined. Erythrocytes were examined using a hemocytometer and then calculated using an improved Neubauer. Leukocyte count and differential leukocytes were analyzed using Giemsa staining [20]. For 1 mL blood, 3–5 fish were required.

### Check blood sugar level

Blood glucose was examined using a blood glucose test kit (GlucoDR Auto AGM-4000, GlucoZen Ltd, Dudley, UK) [21] 24 h post-infection.

### Statistical analysis

The data were analyzed using a one-way analysis of variance, with a 95% confidence level, using IBM SPSS version 27 (IBM Corp., NY, USA). The data were expressed as mean ± standard deviation.

## Results

### Survival rate

In the control group (C), Bath immersion (BI1, BI2 and BI3) group, and intraperitoneal injection (IPI3) revealed a 100% survival rate with no fish death. The concentration of bacteria administered to the bath immersion and IPI3 groups was insufficient to cause disease or death among the fish. The survival rate of the injected fish was higher in the IPI group (22%–100%) than in the IMI group (16%–63%) (Table-1). Both injection methods (IMI and IPI) resulted in higher fish mortality than the immersion method. The higher the bacterial concentration, the greater the mortality rate.

**Table-1:** Effect of applying concentration of *Aeromonas hydrophila* in adult zebrafish.

Treatments	No. of fishes infected	No. of fishes dead	No. of fishes survived	Survival rate (%)	Dead ratio	Survival ratio	Cumulative mortality (%)
IB1	32	0	32	100	0	32	0
IB2	32	0	32	100	0	64	0
IB3	32	0	32	100	0	96	0
IPI1	32	25	7	22	27	7	79
IPI2	32	2	30	94	2	37	5
IPI3	32	0	32	100	0	69	0
IMI1	32	27	5	16	58	5	92
IMI2	32	19	13	41	31	18	63
IMI3	32	12	20	63	12	38	24

In this investigation, the IPI1 group (with a bacterial concentration of 10^11^ CFU/mL) and the IMI groups (IMI1 and IMI2 with bacterial CFS concentrations of 10^9^ and 10^11^ CFU/mL, respectively) had the highest LD_50_ value in the fish incubated within 24 h after infection. LD_50_ was not observed in the BI group, although it was detected in both injection groups.

### Hematological analysis

The infection treatment group with a survival rate >50% continued to undergo blood profile tests. The level of hemoglobin and number of erythrocytes in the control group were higher [6.2 g/dL and 3.64 (10^6^/μL), respectively], but the number of leukocytes was significantly lower [7.27 (10^4^/μL)] compared to the treatment fish infected with *A. hydrophila* bacteria (9.81 × 10^4^/μL–15.31 × 10^4^/μL) (p ≤ 0.05) (Table-2). Hemoglobin and erythrocyte, tended to increase as bacterial concentrations decreased.

**Table-2:** Hematological parameters observed in different treatments at 24 h post-infection.

Treatments	Hemoglobin (g/dL) count	Erythrocyte (10^6^/μL)	Leukocyte (White Blood Cells) count (10^4^/μL)	Lymphocyte (%)	Monocyte (%)	Neutrophil (%)
Control	6.2^ab^ ± 0.0	3.64^b^ ± 0.09	7.27^a^ ± 0.25	83^c^ ± 2.00	5^a^ ± 0.00	12.33^a^ ± 1.53
BI2	6.13^ab^ ± 0.15	1.85^a^ ± 0.08	15.31^c^ ± 0.11	69^b^ ± 1.00	6^a^ ± 0.00	25^c^ ± 1.00
BI3	6.23^b^ ± 0.15	1.92^a^ ± 0.26	13.04^bc^ ± 2.31	72^b^ ± 1.00	8.67^b^ ± 1.53	21^b^ ± 3.46
IPI2	6^a^ ± 0.0	1.45^a^ ± 0.33	14.55^c^ ± 2.25	63.33^a^ ± 1.53	12.67^c^ ± 0.58	24^c^ ± 1.00
IPI3	6.2^ab^ ± 0.0	1.85^a^ ± 0.63	11.43^bc^ ± 1.93	71^b^ ± 0.00	10.67^bc^ ± 0.58	18.33^b^ ± 0.58
IMI2	6^a^ ± 0.0	2.34^a^ ± 0.56	14.94^c^ ± 0.27	64.33^a^ ± 0.00	12.67^c^ ± 0.58	23^c^ ± 0.00
IMI3	6^a^ ± 0.0	2.39^a^ ± 0.16	9.81^ab^ ± 0.10	71.67^b^ ± 0.00	11^c^ ± 1.00	17.67^b^ ± 0.56

Data presentation: mean ± SD. SD=Standard deviation. Different superscripts indicate a significant difference, p < 0.05

The number of leukocytes significantly decreased when the number of bacteria decreased. In the differential leukocyte examination, the number of lymphocytes was higher than those of monocytes and neutrophils in all treatment groups. Based on the variations in bacterial concentrations, the number of lymphocytes in the BI group tended to be greater than that in the IPI and IMI groups.

### Blood glucose testing

The blood glucose levels in the control group did not significantly differ from those in the infected group. Except for the IMI2 (90 mg/dL) and IMI3 (73 mg/dL) groups, the blood glucose levels in the control group tended to be lower (91.67 mg/dL) than those in the other treatment groups (99.67–186.7 mg/dL) following BI groups and IMI groups (Table-3). The blood glucose levels were lower in fish with high mortality rates (IMI2 and IMI3) than in those with low mortality rates (BI1–BI3 and IPI1–IPI2).

**Table-3:** Mean level of blood glucose in all treatment groups.

Treatments	Level of blood glucose (mg/dL)
Control	91.67^a^
BI1	99.6
BI2	99.67^a^
BI3	104.67^a^
IPI1	186.7^a^
IPI2	138.33^a^
IPI3	142.67^a^
IMI1	123.7^a^
IMI2	90^a^
IMI3	73^a^

Similar superscripts indicate a non-significant difference, p < 0.05

## Discussion

Fish infected using the injection and immersion method exhibited death leading to *A. hydrophila* infection, namely, abdominal distension, hemorrhagic ulcers on the body surface, organ rotting, and loss of tails and fins. The fish exhibited decreased appetite before dying. *Aeromonas* spp. infects the fins, integument, and abdomen, causing ulcerative lesions on the integumentary surface [1]. Chandravanshi *et al*. [22] used *Cyprinus carpio* (common carp) fish, which showed clinical signs such as hemorrhagic and rotting tails and fins, lesions at the injection site, loss of skin and scales, abdominal distension, and dropsy. However, the study did not determine whether the fish made abnormal movements and weakened pectoral fin movements, which are signs of approaching death conditions, as observed in a survey conducted by Samayanpaulraj *et al*. [1]. Tabarraei *et al*. [23] demonstrated that fish infected with *A. hydrophila* before death exhibited restlessness, erratic swimming, dark pigmentation, seizures with increased respiratory rate, and excessive mucus production. Nevertheless, in this study, only mucus excess was observed. Fish death was driven by diminished host immunity and virulence factors from *A. hydrophila*. The pathogenicity of *A. hydrophila* bacteria is mediated by virulence factors such as cytolytic heat-labile enterotoxin, cytotonic heat-stable enterotoxin, aerolysin, hemolysin (HLY), elastase, and lipase), which can damage fish body tissues until the fish die [1].

The LD_50_ value indicates the virulence level of the bacteria [24]. Bacteria with an LD_50_ value between 10^4.5^ and 10^5.5^ CFU/mL are very virulent; those with an LD_50_ value between 10^5.5^ and 10^7^ CFU/mL are lethal; and those with an LD_50_ value >10^7^ CFU/mL are avirulent. The results of this study indicated that the *A. hydrophila* bacteria used were avirulent, as their LD_50_ values were >10^7^ CFU/mL. Nonetheless, the mortality rate was extremely high 24 h after the infection.

The use of freshwater fish and zebrafish infected with *A. hydrophila* as animal challenge models for the prevention and treatment of infectious illnesses is quite common. According to a study by Srivastava *et al*. [25], LD_50_ was observed in *Labeo rohita* infected with *A. hydrophila* (2 × 10^7^ CFU/mL) 96 h after infection. According to a study by Rodríguez *et al*. [16], adult zebrafish treated with *A. hydrophila* through IP injection at a bacterial CFS concentration of 10^5^ CFU/mL (bacterial suspension 5 × 10^7^ CFU/mL) reached a 60% mortality rate in 1 day. With a 5 × 10^8^ CFU/mL concentration, they had a 100% mortality rate 1 day post-injection. In another study, zebrafish larvae infected with *A. hydrophila* at a concentration of 10^9^ CFU/mL by bath immersion [26], 3.3 × 10^7^ CFU/mL by IP injection [18], and 10^6^ and 10^7^ CFU/mL by immersion [17] demonstrated mortality.

Based on the natural route of entry of *A. hydrophila*, the investigation employed the bath immersion approach. *Aeromonas hydrophila* bacteria enter the fish’s body through the gills, mouth, eyes, and skin. Due to the probability that the bacteria were avirulent and the concentration was insufficient to enter the fish’s blood vessels, the bacterial concentration was lower in the plasma of fish exposed to the bath immersion method than in those exposed to injection [27]. Under immersion conditions, fish are more susceptible to cold stress, which increases their susceptibility to infection. In this study, however, immersion was preferable to injection for fish survival. This study was identical to one conducted by Sharon *et al*. [28], who demonstrated that exposure to stress did not alter the sensitivity of guppy fish to infection; therefore, the mortality rates were not statistically different from those of healthy control fish. In bath immersion, *A. hydrophila* will be in direct contact with the entry pathway of the fish *via* the water, skin, and gills [7] and with lesions. At the beginning of a bacterial infection, bacteria attach to and destroy the scales. *Aeromonas hydrophila* produces chitinase enzymes during infection to break down the chitin coating (scales covered with chitin) [3, 25]. Damaged scales result in the release of mucus, eradicating the existing microbiota. These modifications can cause paracellular or transcellular translocation of microorganisms. Bacteria enter the venous or lymphatic system [29] and spread throughout all body organs, causing tissue damage and even death. Adult zebrafish infected with *A. hydrophila* by bath immersion in a BHIB growing medium containing *A. hydrophila* bacteria (at a concentration range of 10^6^–10^9^ CFU/mL)/viable bacteria exhibited an LD_50_ within 24 h, according to preliminary studies. Contrarily, neither IM nor IP injections resulted in fish deaths (unpublished data). The preliminary studies, supported by Lü *et al*. [30], used animal models of adult zebrafish infected with *A. hydrophila* at a concentration of 1 × 10^8^ CFU/mL in phosphate-buffered saline (pH 7.4) and mixed with 10 L of water through bath immersion, which demonstrated death at 72 h post-infection.

The study demonstrated lower survival or higher mortality rates in fish injected intramuscularly (IMI group) than in those injected intraperitoneally (IPI group). In the PI methods, bacteria bypass natural physical barriers and integument-associated immune mechanisms associated with the natural route of infection [28], making it easier for bacteria to cause damage compared with the entry of bacteria through IM (IMI group). However, the study did not obtain the same results. According to Samayanpaulraj *et al*. [1], the severity of tissue injury can vary depending on the bacterial concentrations and injection techniques. Udomkusonsri *et al*. [27] demonstrated that in well-vascularized fish muscles, bacteria that enter intramuscularly can modify absorption so that the concentration of bacteria that enter is optimal.

As infectious agents with the same concentration, differences in the type of bacteria (CFS or viable bacteria) resulted in varied fatality rates. Extracellular products (ECPs) were present in CFS bacteria, which increased the likelihood of high virulence and death when injected into the fish’s body. Extracellular products, which are produced by bacteria during reproduction and development, are crucial to bacterial pathogenicity and induce a robust inflammatory response that damages host tissues [31]. The results of this study contradicted those of a study by Rodríguez *et al*. [16] that employed IPs of 10 L CFS suspension containing 10^6^ CFU/mL of *A. hydrophila* in adult zebrafish. They reported that ECP in CFS was not involved in bacterial pathogenicity. The study further demonstrated that the administration of *A. hydrophila* infection, washed and unwashed, did not affect fish death (CFS or viable cells). The immersion procedure will result in death if the zebrafish’s tail is severed before bath immersion, allowing bacteria to enter quickly and causing up to 100% mortality rate within 24 h of infection.

Hematological factors also serve as helpful predictors of infection-related stress as alterations in the blood profile reduce tissue oxygenation [32]. Blood cell response is a vital sign of internal and external alterations in animals [33]. Erythrocytes and hemoglobin are used in the blood to bind oxygen and nutrients [19]. The number of erythrocytes is a very steady index. Thus, the fish body uses a variety of physiological compensatory mechanisms to maintain its number within normal physiological limits [33].

The higher the bacterial concentration, the higher the number of leukocytes, whereas the numbers of hemoglobin and erythrocytes decrease. A significant reduction in erythrocyte parameters (hemoglobin concentration, erythrocyte count, and hematocrit levels) indicates anemia. Fish infected with *A. hydrophila* may develop anemia. One day after the infection, anemia was observed in zebrafish in this investigation. The results of this study differed from those of Bektas and Ayik [12], stating that anemia emerged in Nile tilapia 21 days following infection with *A. hydrophila*. Anemia in fish can be induced by several factors, including toxicity, viral and bacterial infections, and poor nutrition, resulting in alterations to the erythrocyte index and morphology. Due to a reduction in the number of erythrocytes, the amount of hemoglobin in the infected fish decreases. As a result of *A. hydrophila* infection, erythrocytes diminished [2].

The number of leukocytes circulating in the blood determines the health state of fish [34]. Leukocytes represent the body’s non-specific defense against infection. The migration of leukocytes from the spleen to the blood circulation of zebrafish infected with *A. hydrophila*, which induces leukocytosis, results in an increase in total leukocytes that protect the body [2]. On the other hand, leukocytes cause bacteria to secrete HLY toxins, causing ulcers and bleeding on the fish’s skin [35]. Immune cells play a role in eliminating pathogenic bacteria by directing leukocytes to areas of infection or inflammation. So that it can be determined that leukocyte cells will increase in infection conditions.

Because monocytes and neutrophils have a shorter lifespan, these two cell types are in plasma in smaller amounts than lymphocytes. Immune cells destroy bacteria by migrating cells to the inflammation site and are responsible for phagocytosis [36], resulting in bacterial death. When an infection arises, the pathogen-associated molecular patterns of pathogens are recognized by pattern recognition receptors, which then activate non-specific immune cells [37]. Neutrophil cells act as the first line of defense against pathogens during infection by performing the initial phagocytosis of cell debris and dead immune cells. As phagocytic cells in the blood vessels, monocytes grow and differentiate into macrophages before migrating to infected organs. Activated macrophages can contribute to apoptosis by destroying cell debris and dead immune cells for homeostasis [17]. Monocytes and neutrophils also play a role in pathogen neutralization through myeloperoxidase production. Monocytes produce nitric oxide and peroxynitrite, which act as antimicrobials [34].

Lymphocytes play a crucial part in the general and local defensive mechanisms of specific immunity against microbes or foreign proteins. Another study demonstrated that *A. hydrophila*-infected carp (*L. rohita*) had increased lymphocytes, an inflammatory response to pathological circumstances [25]. The increase in lymphocytes triggers effector functions in Fc Receptors (FcRs) bearing cells in particular lymphocytes. The role of this receptor is in modulating CD4+ T cell responses. In addition to binding to antibody fraction Fc, surface Fc receptors activate peritoneal macrophages, which kill bacteria by binding to target-coated cells [4].

In fish, stress can interfere with immunity and the regular function of the body’s barrier, allowing pathogens to invade. The physiological responses of fish to stress occur in three stages: changes in circulating cortisol and catecholamine levels, glucose, lactate, ions, glycogen levels, and heat-shock proteins, as well as growth, disease resistance, and behavioral changes. The blood glucose levels of stressed common carp (*Cyprinus carpio* L.) were nearly double those of unstressed fish. Blood glucose and cortisol levels are common stress indicators [29]. Increased blood glucose is usually caused by stress or infection [37]. Induction of *Salmonella* lipopolysaccharide decreases glucagon-like peptide 1 production and increases Homeostatic Model Assessment for Insulin Resistance, leading to glucose regulation [38]. Plasma cortisol is the central molecule that increases in response to stress. In doing so, it causes various secondary physiological reactions, including a rise in blood glucose, the primary source of energy for tissues [32].

This study demonstrated that the MI group had much lower blood sugar levels than the BI and IPI groups. Increased blood glucose levels are not necessarily positively connected with high fish mortality rates related to *A. hydrophila* infection, as the bacterial infection may produce reversible pancreatic cell repair and fast cell recovery [39]. Fish have poor control over their blood glucose levels; even cod can survive with plasma glucose levels close to zero. *In vivo* or *in vitro*, various organs of *rainbow trout* fish serve as glucose soars in response to short-term increases or decreases in glucose levels (within hours). These tissues consist of brain regions such as the hypothalamus, hindbrain, and Brockmann bodies, a unique cluster of pancreatic endocrine cells near the gall bladder [40]. Omnivore fish can regulate blood glucose concentrations within hours to maintain glycemic homeostasis, as observed in *Colossoma macropomum* fish [34].

*Aeromonas hydrophila* colonization in the gut can change the composition of the gut microbiota of adult zebrafish, thereby promoting the development of pathogenic bacteria and reducing beneficial gut bacteria [7]. Proteins produced by the bacterial microbiota in the gut can trigger pancreatic cell growth. The high number of pancreatic cells in zebrafish depends on the number of bacteria and microbial secretion products in the water, even though they are kept in non-sterile water. A study conducted by Hill *et al*. [41] showed that CFS from *A. hydrophila* stimulated the growth of pancreatic cells in 4-day-old germ-free zebrafish larvae. Zebrafish have pancreatic cell development like mammals. The development of insulin-producing pancreatic cells is in line with the growth of gut microbes — the hormone insulin functions to maintain blood glucose levels [39].

This study showed that adult zebrafish could serve as animal models for *A. hydrophila* infection based on changes in blood profiles, blood glucose levels, and mortality rates. The fish survival rates were greater when immersed in water than when injected with bacteria. The number of erythrocytes and hemoglobin decreased in bacteria-infected fish, whereas the number of leukocytes increased. The IM infection of adult zebrafish with *A. hydrophila* at an LD_50_ >10^7^ CFU/mL for 1 day can be employed as a challenge test, offering a repeatable route of infection. However, if the aim is to determine the level of morbidity resulting from a bacterial infection, concentrations can be observed in <24 h. It was impossible to conclusively determine whether the methods and concentrations cause changes in blood sugar levels; therefore, additional research is warranted to demonstrate the potential injury to fish pancreatic cells. In this investigation, *A. hydrophila* infection manifested as abdominal distension and ulcers on the body surface and fins, followed by a mortality rate corresponding to an injection infection. In this study, a histopathological test was not conducted, indicating the presence of tissue damage due to bacterial infection using the three methods. In addition to the dose and route of infection, genetic differences between fish species and susceptibility to disease-causing substances can influence the susceptibility of fish to infection [28].

## Conclusion

Based on the observations of the mortality rates, blood profiles, and blood sugar levels of the infected and healthy groups, adult zebrafish can be used as animal models for *A. hydrophila* infection. The infection of adult zebrafish with *A. hydrophila* at concentrations of more than 10^9^ CFU/mL through IM for 1 day can be employed as a challenge test, which is a more repeatable method of infection than bath immersion.

## Authors’ Contributions

DQS, SS, MM, and HK: Designed the study, statistical analysis, and drafted the manuscript. LEF, AA, and LTS: Statistical analysis and drafted the manuscript. All authors have read and approved the final manuscript.
